# The Biomimetic System of Oleanolic Acid and Oleic Acid at the Air-Water Interface–Interactions in Terms of Nanotechnology-Based Drug Delivery Systems

**DOI:** 10.3390/membranes12121215

**Published:** 2022-12-01

**Authors:** Martyna Krajewska, Katarzyna Dopierała, Krystyna Prochaska

**Affiliations:** Institute of Chemical Technology and Engineering, Poznan University of Technology, Berdychowo 4, 60-965 Poznań, Poland

**Keywords:** oleanolic acid, oleic acid, mixed monolayer, phase separation, excess free energy of mixing, dilatational rheology, interface stability, biomimetic system, phytomedicine

## Abstract

Oleanolic acid (OLA) and oleic acid (OA) are ubiquitous in the plant kingdom, exhibiting a therapeutic effect on human health, and are components of novel pharmaceutical formulations. Since OLA has limited solubility, the utilization of nanotechnology-based drug delivery systems enhancing bioavailability is highly advantageous. We report on the interfacial behavior of the OLA:OA system at various molar ratios, using the Langmuir technique to assess the dependence of the molar composition on miscibility and rheological properties affecting film stability. Specifically, we evaluate the interfacial properties (morphology, thermodynamics, miscibility, and viscoelasticity) of the OLA:OA binary system in various molar ratios, and indicate how the OLA:OA system exhibits the most favorable molecular interactions. We apply the Langmuir monolayer technique along with the complementary techniques of Brewster angle microscopy, dilatational interfacial rheology, and excess free energy calculations. Results demonstrate that the properties of mixed monolayers depend on OLA:OA molar ratio. Most of the systems (OLA:OA 2:1, 1:1, 1:5) are assumed to be immiscible at surface pressures >10 mN/m. Moreover, the OLA:OA 1:2 is immiscible over the entire surface pressure range. However, the existence of miscibility between molecules of OLA and OA in the 5:1 for every surface pressure tested suggests that OA molecules incorporate into the OLA lattice structure, improving the stability of the mixed film. The results are discussed in terms of providing physicochemical insights into the behavior of the OLA:OA systems at the interface, which is of high interest in pharmaceutical design.

## 1. Introduction

Phytomedicine-based therapies utilize naturally occurring chemical substances from various plant species. Herbal constituents are extracted from all the plant organs, such as roots, leaves, stems, flowers, seeds, or even the by-products (gums, resins). Although phytomedicine has been practiced for thousands of years, in the context of modern trends in pharmaceutical formulations, it is gaining new meaning. Recently, considerable attention has been paid to the development of novel drug delivery systems for herbal drugs. The new approaches are based on nanocarriers such as micro- and nanoemulsions, nanoparticles, matrix systems, liposomes, and solid dispersions. Compared to conventional therapies, nanophytomedicine brings numerous benefits, the most important of which are better therapeutic efficacy, improved surface-area-to-volume ratio, and stability of sustained drug release, as well as enhanced solubility and bioavailability of the active substance [1,2,3,4]. This latter feature is particularly desirable in the case of triterpenoid compounds such as oleanolic acid.

Oleanolic acid (OLA) is the common name for 3β-hydroxyolean-12-en-28-oic acid. It is a naturally occurring pentacyclic triterpenoid found in the leaves and roots of *Olea europaea*, *Aralia chinensis* L., *Viscum album* L., and many other plant species. Within the OLA molecule, between the 12th and 13th carbon atom, there is one unsaturated bond in its structure and two polar groups: a carboxyl group and a hydroxyl group substituted to the 3rd carbon atom in the β position. OLA is considered to be a bolaamphiphilic compound—it has two hydrophilic regions located at the opposite sites of the molecule. The polar regions are separated by the hydrophobic part of the molecule, comprising five tightly connected cyclic rings. The presence of two polar groups should enhance the solubility in water; however, in the case of OLA, it is highly limited since the significant rigidity of the pentacyclic fragment disables molecular bending. Thus, one molecule cannot contact both polar groups with the water phase or reach a U-shape conformation at the interface. Consequently, OLA creates insoluble monolayers, where molecules can be oriented in one of two possible ways: orientation A—if the hydroxyl group is in contact with the aqueous phase, then the molecules are oriented perpendicularly to the interface, and orientation B—where the carboxyl group has contact with the subphase and molecules are tilted towards the water [5,6,7,8,9,10]. Within one monolayer, there are molecules with both orientations, so OLA monolayers are inhomogeneous.

OLA brings promising biological effects due to its antitumor, anti-inflammatory, antiviral, antioxidant, cardioprotective, and hepatoprotective actions. The biotherapeutic efficacy of the triterpenoid molecules can be attributed to the presence of the electron donor atoms participating in the formation of coordination bonds with d-block metal atoms. Unfortunately, the applicability of oleanolic acid has been limited because of the poor water solubility and low bioavailability by oral administration [3,11,12,13,14,15]. Conventional formulation types for OLA are tablets and capsules; however, various nanotechnology-based drug delivery systems are under investigation. Current considerations are focused on the stability and oral absorptivity increase, as well as on the improvement of the pharmacokinetic characteristics [13].

One of the strategies to enhance the in vivo efficacy of OLA is encapsulation by nanostructured lipid carriers (NLCs). Zhang et al. [16] used NLCs to entrap gentiopicrin and oleanolic acid simultaneously using a film–ultrasonic method. In this system, the oil phase is comprised mainly of oleic acid. Another innovative approach is a self-microemulsifying drug delivery system (SMEDDS), which provides the drug release in a sustained manner. One of the considered vehicles for oleanolic acid is oleic acid. Other innovative OLA formulations include nanoparticles, nanocapsules, nanospheres, nanoemulsions, liposomes, solid lipid nanoparticles (SLN), and self-emulsifying drug delivery systems (SEDDS). Moreover, olive oil enriched with oleanolic acid is considered a functional food due to its anti-inflammatory activity and ability to prevent metabolic syndrome. OLA-enriched olive oil is also intended for pharmaceutical composition formation, together with excipients, adjuvants, carriers, or even drugs and active substances [3,9,10,12,17,18].

The aforementioned oleic acid (cis-9-octadecenoic acid, 18:1, OA) is classified as a long-chain monounsaturated fatty acid. OA presents significantly different behavior at the interface. The molecule is comprised of 18 carbon atoms in the chain and contains a C=C double bond of *cis* configuration located between 9th and 10th carbon atoms. This double bond affects the flexibility of the hydrocarbon chain, hence influencing the molecule’s alignment and the fluidity of the interfacial film. Compared with the saturated analog (stearic acid, 18:0), the chain flexibility of OA is significantly limited. Thus, the intermolecular adhesion decreases and the limiting area is enlarged due to the lower possible molecular packing.

OA is a commonly used pharmaceutical excipient included in various dosage forms. It acts as a solubility enhancer in the gastrointestinal tract delivery systems, an emulsifying agent in topical pharmaceutical formulations, and a penetration enhancer in transdermal formulations, and it contributes to ensuring product stability [19]. A considerable amount of OA is present in natural oils including olive, sunflower, rapeseed, peanut, and palm oils. It is worth emphasizing that olive oil, besides monounsaturated fatty acids (mainly OA), also contains a significant amount of OLA. Moreover, OLA is perceived as one of the factors contributing to the beneficial effects of olive oil consumption on the human organism [12,20,21].

A separate area of the natural occurrence of OLA and OA constitutes wax covering plant leaves and fruits. Together with esters, fatty acids, fatty alcohols, and n-alkanes, OLA is the main component of these waxes, protecting plants against diseases, insects, fungi, and excessive loss of water [7,22,23].

The biomimetic system of OLA and OA acts as the bioavailability-improving agent in pharmaceutical formulations containing other active substances like curcumin. The composition retarding the onset of Alzheimer’s disease symptoms was reported previously in a US patent. The proposed dietary supplement comprises curcumin, piperine, oleic acid, oleanolic and ursolic acids, galantamine, and huperzine. The supplement is intended for patients in the early stages of the disease and is to be administered orally. The mixture is of therapeutic potential due to the increased gastrointestinal absorption of the curcumin into the bloodstream, which can delay the onset of the symptoms of Alzheimer’s disease. Oleanolic acid combined with ursolic acid and oleic acid were reported to increase the bioavailability and absorption of the curcumin and other nutrients [13,24].

Colloidal carriers ensure a favorable environment for the active molecules and provide a matrix responsible for stable formulations. Extensive investigations are needed for the development of oily excipients in novel drug delivery systems. The good performance of those systems determines the product’s utility and efficiency [25,26]. Identifying factors that influence the nanoformulation’s properties is a significant step in designing novel drug delivery systems [16]. Since the thermodynamic stability of oily excipients is usually unknown, we offer an extensive physicochemical analysis of the exemplary system inspired by nature. Differences in the molecule’s structure and properties are the crucial issues in the Langmuir monolayer stability, which could be transferred to pharmaceutical products. Moreover, for the multi-component structures, the interactions between substances should be considered, as the repulsive interactions may even lead to phase separation, which reduces the product efficiency.

In this paper, we explore the physicochemical properties of the biomimetic system of OLA and OA at the air/water interface in terms of its stability in pharmaceutical formulations. OLA-OA mixed systems in various molar ratios were examined for the miscibility and excess free energy of mixing as well as the rheological characteristics.

## 2. Materials and Methods

### 2.1. Materials

The ultrapure water (18 MΩ·cm, 71.98 ± 0.01 mN/m, pH 6.25) used as a subphase was from the PureLab Classic UV system (ELGA, High Wycombe, UK). The film-forming substances in the experiments were oleanolic acid (3β-hydroxyolean–12-en–28–oic acid; 97%, OLA) and oleic acid (cis-9-octadecenoic acid, 99%, OA), purchased from Sigma-Aldrich (St. Louis, MO, USA). Chloroform Uvasol of high purity (from Merck, Darmstadt, Germany) was used for the preparation of spreading solutions.

### 2.2. Langmuir Experiments

The mixtures of OLA and OA at various molar ratios (1:5, 1:2, 1:1, 2:1, and 5:1) were dissolved in chloroform. All Langmuir experiments were conducted using a Teflon trough of the surface area equal to 273 cm^2^. The trough by KSV NIMA (Helsinki, Finland) was equipped with two movable barriers of Delrin to compress and expand monolayers. A F12 circulator (Julabo, Seelbach, Germany) maintained the subphase temperature during the experiments at 25 ± 0.1 °C. The barrier speed during the compression and expansion was 10 mm/min. Every process was conducted at least 3 times to confirm the reproducibility of the results. Spreading solutions (in individual experiments—oleanolic acid, oleic acid, or mixtures dissolved in chloroform) were carefully applied on the water subphase using the Hamilton microsyringe. After solvent evaporation (at least 10 min), the monolayer was compressed until collapse. The surface pressure (π) and the area per molecule (A) were recorded and performed as the π–A isotherm.

The compression modulus Cs^−1^ provides information about the physical state of the monolayer, as well as about compressibility and arrangement of molecules within the monolayer. It was calculated based on the π–A isotherm data for each of the solutions according to Equation (1).

(1) $${\mathrm{Cs}}^{-1}=-A{\left(\frac{d\pi }{\mathrm{dA}}\right)}_{p,T}$$

Relaxation experiments were conducted by a step change to a specified surface pressure π. The system than relaxed through changes in the surface area necessary to maintain that surface pressure. The software recorded the area per molecule (A) changing in time (t). For ease of comparison, the results on the graphs are recorded as the relative area (A/A_0_), where A_0_ is the initial area per single molecule when the desired surface pressure is reached.

### 2.3. Analysis of Miscibility

The area per molecule in a mixed monolayer A_12_ at the given surface pressure can be calculated based on the π–A isotherm data and plotted as a function of the mixed monolayer composition. In the case of the ideal binary system, the area per molecule is simply (Equation (2)):

(2) $$A_{12}{}^{\mathrm{id}}=A_{1}X_{1}+A_{2}X_{2}$$

where A_1_ and A_2_ are molecular areas of the respective components in the pure monolayers at the given surface pressure; X_1_ and X_2_ are molecular fractions of substances creating a film.

For a real mixed monolayer system, however, the measured area per molecule A_12_ is taken to be a complex function (Equation (3)):

(3) $$A_{12}=f\left(X_{1},X_{2}\right)$$

The excess free energy of mixing $\Delta G^{\mathrm{exc}}$ was calculated according to Equation (4) and plotted as the function of monolayer composition.

(4) $$\Delta G^{\mathrm{exc}}=N\overset{\pi }{\underset{0}{\int }}\left(A_{12}-X_{1}A_{1}-X_{2}A_{2}\right)d\pi$$

where N states for the Avogadro’s number.

### 2.4. Brewster Angle Microscopy

BAM images were captured by the MicroBAM (KSV NIMA, Helsinki, Finland) when performing the π–A isotherms. Images were retrieved during the compression and expansion of the monolayer using identical settings for every system tested (to enable image comparison). The size of the image is 3.6 × 4.0 mm.

### 2.5. Dilatational Rheology Studies

Oscillation of the barriers of a Langmuir trough produces a superposition of pure dilatation and two-dimensional extension. However, the second contribution is negligible, so we approximated barrier motion as a pure dilatation. The dilatation rheology measurement consisted of consecutive oscillation and relaxation periods to assess the viscoelastic properties of the film in time. The measurement was initiated with compression of the monolayer to a desired surface pressure. After 1 min, the barrier oscillation was initiated and followed by a 10-min relaxation period prior to a subsequent oscillation. The experiment was continued until 15 oscillation slots were conducted or until monolayer collapse (the methodology of the experiment is additionally depicted in Figure S1 in the Supplementary Materials). Dilatational rheology experiments were performed at 5 mN/m with a barrier speed of 10 mm/min, frequency of 50 mHz, and area change of 1%.

## 3. Results

### 3.1. The Structure of OLA-OA Binary Monolayers

**Figure 1:**
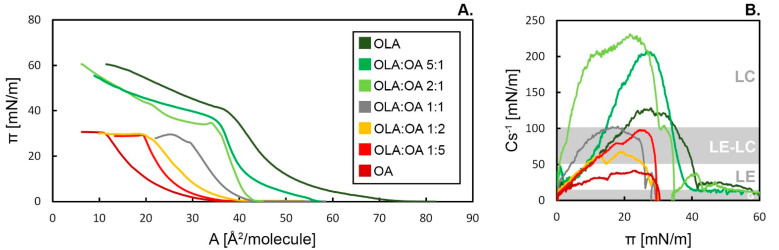
The π–A isotherms of pure substances and mixed OLA:OA systems (**A**) and the compression modulus vs. surface pressure graph (**B**) based on the isotherms, with the ranges of the compressibility modulus states highlighted and described. Plots illustrate the extremely various morphology of OLA and OA monolayers, as well as characteristic features of binary systems altering with the monolayer composition. Some systems (OLA:OA 2:1, 1:1, and 1:2) appear unstable at high surface pressure, which may indicate phase separation.

The surface pressure (π, mN/m) versus area per molecule (A, Å^2^/molecule) dependence was determined for pure OLA and OA monolayers and mixed (OLA:OA 5:1, 2:1, 1:1, 1:2 and 1:5) systems and are depicted in Figure 1A. The isotherm-specific parameters (such as A_lift-off_, A_lim_, or π_coll_, see Figure S2) are summarized in Table S1 in the Supplementary Materials. Conducted isotherm experiments confirm that both pure and mixed systems create insoluble monolayers at the air/water interface, and every individual system shows features that strongly depend on the film composition. The shape of the isotherms of OLA and OA reflects the extremely different molecular structures of these chemical substances. It can be seen that the A_lift-off_ value for the OA monolayer is equal to 34 Å^2^/molecule and 75 Å^2^/molecule for the film of OLA. Upon the monolayer compression, the surface pressure increases as the mean area per molecule decreases, until reaching the π_coll_ value of 31 mN/m for OA, which corresponds to the monolayer collapse. The case of the oleanolic acid monolayer is more complex. Due to the specific molecule structure, oleanolic acid creates a rigid film at the interface, and the monolayer exhibits stability up to 10 mN/m. The presence of the hydrophilic hydroxyl and carboxyl groups on the opposite sites of the molecule, regardless of the arrangement of molecules at the air/water interface, makes the assembly energetically unfavorable. Thus, above 10 mN/m, the OLA π–A isotherms are not repeatable, due to instabilities of the molecule’s orientation and structure [5,6,7,27]. The collapse of the OLA monolayer starts at π_coll_ value of 41 mN/m.

The shape and position of the π–A isotherms of mixed systems are clearly dependent on the monolayer composition. The curves of mixed systems are located between the pure OA and OLA isotherms. Increasing the content of OA shifts the mixed monolayer isotherms towards a lower molecular area corresponding to values reminiscent of fatty acid isotherm. The systems where OLA is the dominant constituent over OA, especially OLA:OA 5:1, are qualitatively similar to the isotherms of pure OLA monolayer. On the other hand, the systems with excess concentrations of OA (OLA:OA 1:2 and OLA:OA 1:5) and equimolar systems do not exceed the π_coll_ value of pure OA isotherm. However, the observed fluctuations of OLA:OA 2:1, 1:1 and 1:2 curves reveal signs of instability (double collapse regions, disrupted isotherm shape). In fact, two collapse regions can be distinguished in these systems, which are 30 mN/m and 35 mN/m, 25 mN/m and 29 mN/m, 27 mN/m and 29 mN/m, respectively. This phenomenon is associated with a lack of miscibility among mixed monolayer components [28,29,30]. In the case of a monolayer of miscible components, the value of the collapse surface pressure is in the range of π_coll_ for pure monolayers. Otherwise, when the monolayer components do not mix with each other or the miscibility is limited, two collapse regions may occur within the course of the π–A isotherm, which indicates the phase separation of the mixed monolayer. The miscibility aspects of the binary systems will be discussed in detail further in the paper.

The molecules conformation at the interface and the interactions between OLA and OA molecules are reflected in the compression modulus graph [Figure 1B]. The Cs^−1^ is strongly related to the isotherm course and molecule’s orientation. The more rapid the surface pressure increase, the higher the compression modulus is; thus, a higher monolayer compression is possible.

Due to the presence of an unsaturated bond within the hydrocarbon chain, the OA monolayer does not exceed the value of Cs^−1^ = 50 mN/m; thus, referring to the study of Rideal and Scott [31], it remains in a liquid-expanded (LE) state. The unsaturation enhances the monolayer fluidity and prevents the molecules from being tightly compressed.

The slope of the OLA isotherm changes up to a value of π = 10 mN/m and becomes steeper, which influences the Cs^−1^ values. In the G and LE phases, orientation B of the OLA molecule is more favorable (tilted above the air/water interface). Based on the molecular dimension, the area occupied by a single molecule is estimated as ca. 72 Å^2^, which corresponds to the value of OLA A_lift-off_. Above a surface pressure value of 10 mN/m, the monolayer undergoes an LE–LC phase transition, and orientation A starts to predominate within the monolayer. For nearly vertical OLA orientations, with the main axis towards the air/water interface, the molecular area value equals ca. 40 Å^2^, which, in turn, corresponds to the value of mean area per molecule at the collapse of the monolayer, where the molecules are packed the tightest. Taking this into account, as well as the value of A_lim_ of OLA monolayer, it can be concluded that in liquid states of the monolayer both of the OLA molecule orientations coexist. According to the phase classification, the OLA monolayer at the maximal compression is in the LC phase.

As can be seen from Figure 1B, the compressibility of mixed systems is strongly dependent on their composition. The mixed systems with excess OLA concentrations reach Cs^−1^_max_ values higher than the pure OLA monolayer. Furthermore, the values of Cs^−1^_max_ indicate that the mixed compositions of OLA:OA 5:1, 2:1 and 1:1 reach the LC thermodynamic state, while OLA:OA 1:5 and 1:2 are on the line between LE and LC states. It can be noticed that even a small amount of OLA in the OA monolayer causes a condensing effect and enhances the compressibility of mixed monolayers.

Analysis of the Brewster Angle Microscopy images provides evidence for the phase separation within the compressed monolayers. BAM was performed for both pure and mixed monolayers of OLA and OA during the π–A isotherm compression/expansion cycles. In the images, the very dark regions correspond to the water subphase, while the monolayers are visible as gray areas of various brightness depending on differences in orientation-induced monolayer thickness. The brighter the region, the thicker the monolayer is.

**Figure 2:**
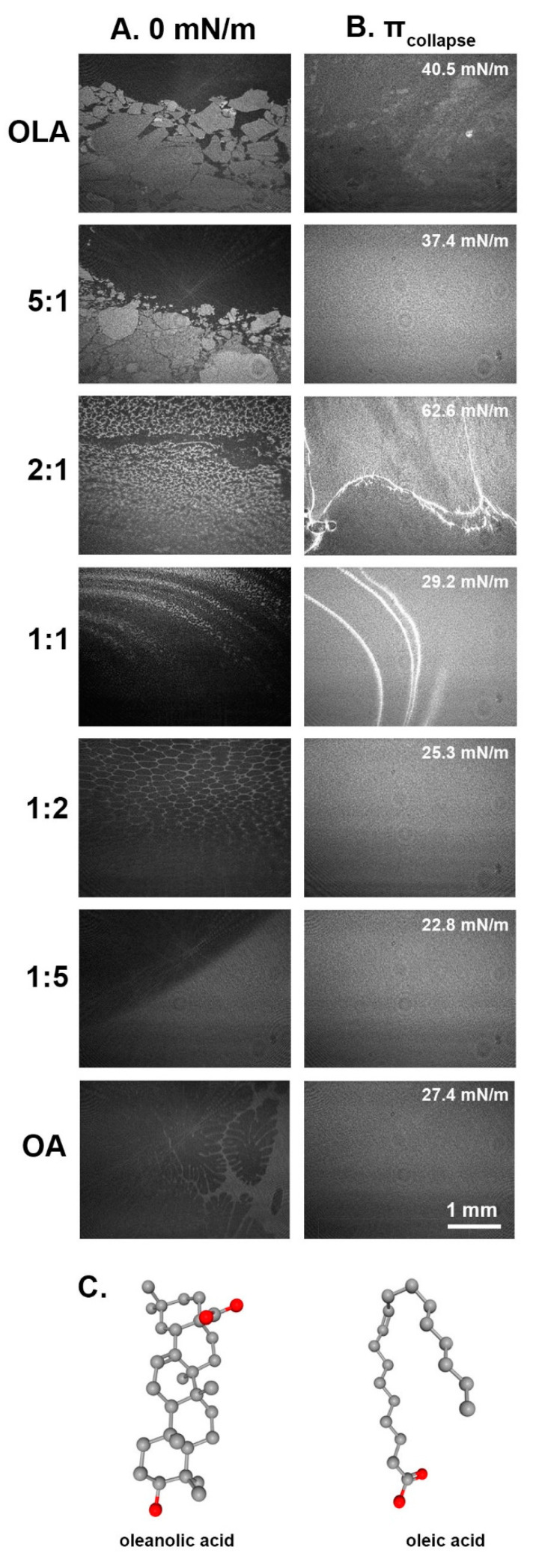
The BAM images of pure OLA and OA monolayers, as well as binary systems of OLA:OA for various mole fractions (**A**) at 0 mN/m just before compression start and (**B**) at π close to collapse, specific for a particular system. The mixed monolayers are of intermediate morphology between pure systems, but in the case of OLA:OA monolayer, there are some bright domains demonstrating phase separation. Importantly, a small content of OA to OLA monolayer (OLA:OA 5:1) resulted in a homogenous monolayer morphology. (**C**) Chemical structure depiction of oleanolic acid (left) and oleic acid (right).

Figure 2 depicts obvious differences in the monolayer structures of pure OLA and OA. Within the OLA monolayer, there are angular, tile-like domains of various size and brightness. This fact may correspond with the coexistence of the two orientations of the OLA molecule in the monolayer. There is a difference in the OLA film thickness depending on which polar moiety of the molecule is anchored in the water subphase. Therefore, the monolayer domains of diverse brightness can be observed. Although upon the monolayer compression, domains are fused (at ca. 29.73 mN/m), the surface inhomogeneity (brighter regions and wrinkles) can be observed during the whole process.

On the other hand, at the air/water interface, OA forms characteristic domains of rounded shape (so-called “foam-like” structures). These microdomains are clearly seen for a relatively short time during the monolayer compression because a slight increase in surface pressure due to the compression leads to the fusion of the microdomains. The OA monolayer is homogenous even at surface pressures close to π_coll_ (ca. 27.43 mN/m). The binary monolayers exhibit intermediate features between the morphology of pure substances. The mixed systems with the excess of OA correspond to the pure OA monolayer and do not show indications of collapse or phase separation when compressed. The system of OLA:OA 5:1 reveals an analogous morphology as pure OLA, but the edges of the tiles are smoother. But, more importantly, in contrast to OLA after compression, the OLA:OA 5:1 monolayer morphology is uniform, devoid of domains or aggregates. However, higher OA content in the OLA monolayer (OLA:OA 2:1 and 1:1) leads to lace-like structures at the interface, followed by the bright, elongated aggregates demonstrating phase separation.

### 3.2. Interactions among Monolayers–Thermodynamic Analysis of the Miscibility

**Figure 3:**
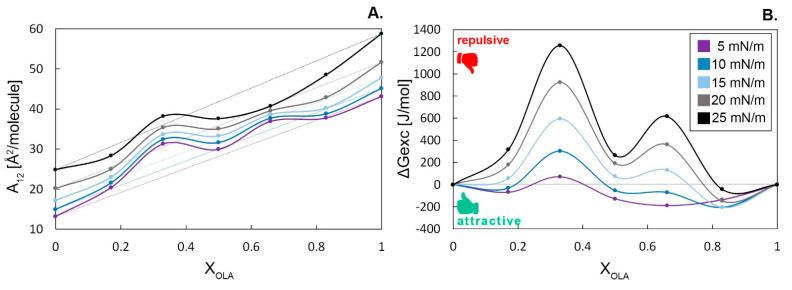
(**A**) The plot of mean area per molecule (A_12_) vs. composition (X_OLA_) for OLA:OA mixed monolayers and (**B**) the excess free energy of mixing (ΔG^exc^) vs. composition (X_OLA_) for OLA:OA mixed monolayers; both are at constant surface pressures ranging from 5 to 25 mN/m. The gray lines in figure A represent the behavior of the ideal binary system. Due to the positive values of ΔG^exc^ at B, the system of OLA:OA 1:5 seems to be immiscible with repulsive interactions between components, in contrast to the OLA:OA 5:1 system, where components are miscible in a whole range of surface pressures investigated.

The miscibility of a binary Langmuir monolayer is determined by the interactions between its components. Analogous to mixtures in bulk systems, film components can be immiscible, partially miscible, or completely miscible. If the monolayer components are fully immiscible or their mixture is ideal, the dependency of the mean area per molecule on the mixed system composition is linear [Figure 3A]. However, mixed monolayers usually exhibit a non-ideal behavior due to the interactions between their components. Positive deviation evidences the presence of repulsive interactions, while negative deviations indicate attractive interactions between the components. Positive deviation values can also indicate phase separation within the binary monolayer [28].

The thermodynamic analysis of the mixed systems as a graph of the excess free energy of mixing ΔG^exc^ vs. the molar fraction of OLA (X_OLA_) is shown in Figure 3B. A monolayer composed of perfectly miscible substances exhibits a ΔG^exc^ value of zero. The deviations plotted in Figure 3B indicate the presence of various interaction types between the components depending on the composition. The negative values of ΔG^exc^ signify stronger attraction compared with repulsion between the binary monolayer components in comparison to single-component monolayers. Moreover, the more negative the value, the more pronounced the stability of the mixed monolayer. On the other hand, the positive value of ΔG^exc^ indicates weaker attraction (stronger repulsion) between the molecules in the binary monolayers compared with pure substances.

Strong evidence of the phase separation in the OLA:OA 1:2 system was found. The positive values of ΔG^exc^ reached by the mixed system OLA:OA 1:2 in the whole range of surface pressure values indicate the presence of strong repulsive interactions between the components. The same behavior can be noticed in the case of the systems OLA:OA 1:5, 1:1, and 2:1 at π value above 10 mN/m. However, within a relatively loosely packed monolayer (at 5 and 10 mN/m), those systems are miscible. On the other hand, the binary system OLA:OA 5:1 exhibits the negative values of ΔG^exc^ in the whole range of surface pressures tested. It is most likely that in this system, attractive interactions between the molecules occur, which may cause relatively good miscibility of the monolayer components. What is more, sufficiently low values of ΔG^exc^ support the stability of the monolayer in time. This observation may find similarities in the analysis of the relaxation measurements.

### 3.3. Mixed Monolayer Stability Investigated by Relaxations

**Figure 4:**
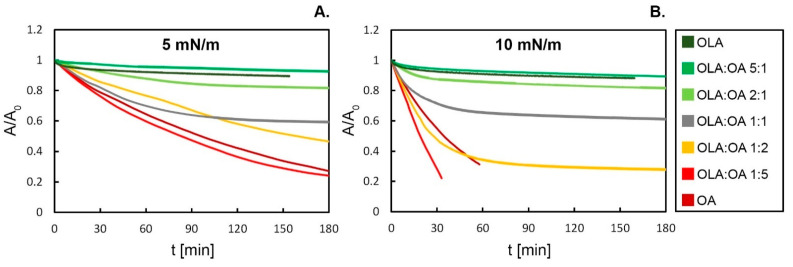
The relative area changes during the relaxation experiment for single substance monolayers (OLA and OA), as well as for binary monolayers for various mole fractions at 5 mN/m (**A**) and 10 mN/m (**B**). In general, mixed OLA:OA systems are characterized by the intermediate stability between the extremely different monolayers of pure substances. However, a system with a small addition of OA to OLA is more stable than OLA itself, and a system with a small addition of OLA to OA degrades even faster than a pure OA monolayer.

The stability of the monolayers over time was assessed based on the relaxation measurements at constant surface pressures of 5 and 10 mN/m, due to the stable assembly of OLA at the interface in this π range. The dependence of the relative molecular area A/A_0_ as a function of time for both values of π is presented in Figure 4. The rates of monolayer disruption of pure OLA and OA differ significantly for both levels of tested surface pressures. For the OLA monolayer, the relative surface area decreased by only 10% and 12% in 150 minutes for 5 and 10 mN/m, respectively. The relaxation curve of OLA at both values of surface pressure decreases in the initial stage of the measurement, but it stabilizes after ca. 30 min. Due to the unsaturated bond in the hydrocarbon chain, the stability of the OA monolayer at the interface is markedly limited—in 150 min, A/A_0_ dropped by 67% at 5 mN/m, and at 10 mN/m, the monolayer existed at the interface only for 60 min. It has been found that the stability of the binary system depends on the monolayer composition. For both surface pressures investigated, the tendency in the disruption kinetics is quite consistent. The system of OLA:OA 5:1 exhibits a relaxation curve at an even higher A/A_0_ level than the pure OLA monolayer, contrary to OLA:OA 1:5, where the addition of a small amount of triterpenoid to the oleic acid monolayer accelerates the disruption of the monolayer. Bearing in mind the miscibility of the mixed systems analysis, the enhanced stability of OLA:OA 5:1 is likely caused by the presence of attractive interactions between the monolayer components, while the repulsive interactions within OLA:OA 1:5 causes the stability decrease. For the systems of OLA:OA 2:1, the relaxation curves correspond to the pure OLA monolayer graph. The disruption of this system is relatively low in comparison to the other mixed systems, and even the stabilization of A/A_0_ occurs. The course of the equimolar system’s relaxation also stabilizes, but at lower values of A/A_0_ and after a relatively long time. The system of OLA:OA 2:1 exhibits analogous features to the relaxation curve of oleic acid, but at a higher relative surface area. What is more, at π = 10 mN/m this system achieved a constant A/A_0_ value.

### 3.4. Dilatational Rheological Properties of the Mixed Systems

**Figure 5:**
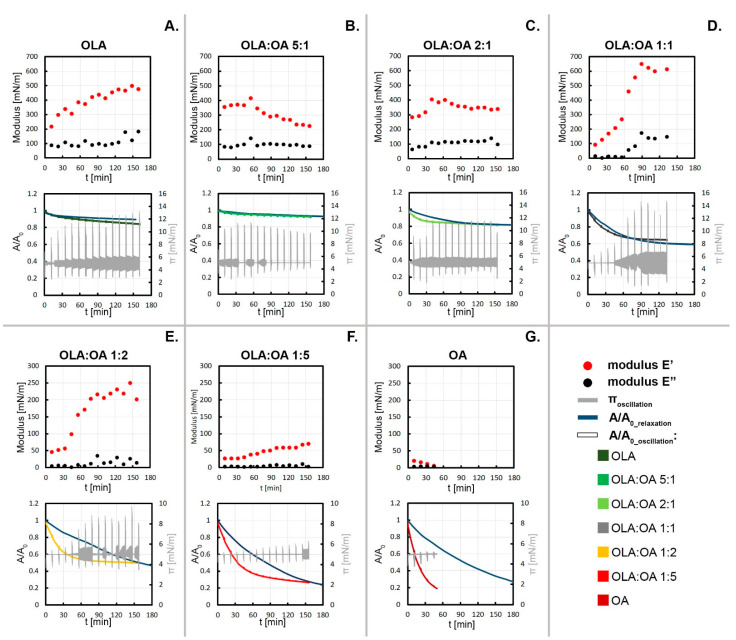
Viscoelastic moduli E’ and E’’ of the pure systems (**A**,**G**) and binary monolayers of OLA and OA (**B**–**F**) plotted vs. time and complemented by the graphs of the relaxation during oscillation experiments and without disturbances separately, as well as surface pressure vs. time, varying significantly due to the oscillation. Such a comprehensive set of results allows for a holistic assessment of system behavior at the interface due to the application of an oscillating force to the monolayers.

The rheological properties of the therapeutic system play a significant role in the selection of the appropriate route of administration of medicinal substances to the human body [32]. Thus, the viscoelastic properties become an important consideration of the physicochemical studies of the OLA:OA binary system. The dilatation rheology measurements were performed using the barrier oscillation method to follow the relaxation processes. The elastic dilatational modulus E’ and dilatational viscous modulus E’’ of each pure and mixed system are plotted over time t [Figure 5, top panels]. The rheological data are supported with the relaxation plots to track the changes in viscoelastic and thermodynamic properties simultaneously [Figure 5, lower panels]. Oscillations were repeated cyclically every 10 minutes. Relaxation data were recorded during the oscillation itself, as well as during the waiting time, when π was kept constant at 5 mN/m [Figure S1 Supplementary Materials]. We observed the asymmetry of the oscillation peaks towards the desired value [Figure S3 Supplementary Materials], which is in line with the feature attributed to the dilatational rheology. In the dilatation strain, the compression step induces a quantitatively different outcome than the expansion step leading to a different response in surface pressure values. According to the fact that the force of the oscillating barriers applied to the monolayer causes a monolayer degradation (at a rate depending on the composition), the relaxation curves without additional stress were added for a broader view. Moreover, we also present the values of the surface pressure vs. time during the oscillation/relaxation experiment, because it was noted that barrier movement causes enormous variations in surface pressure for systems with substantial OLA content.

From Figure 5, it can be seen that the values of elastic modulus (E’) are higher than viscous modulus (E’’), which means that both OLA and OA monolayers exhibit more elastic than viscous properties. However, despite the similar dependence for pure substances, the values of the viscoelastic moduli and alterations over time differ significantly. E’ and E’’ rate changes are associated with the physicochemical properties of the film. Kinetics of the A/A_0_ decrease (as an effect of the oscillation) is characteristic of specific film compositions. It is evident that the relaxation behavior is highly sensitive to film composition.

In the case of pure OLA monolayers, rapid decreases in A/A_0_ leads to an increase in the elastic modulus whereas the viscous modulus remains almost unchanged at 100 mN/m [Figure 5A top panel]. The maximal value of E’ was 500 mN/m after 150 min. Only in the last stage of the measurement does the viscous modulus grow. The difference between the course of the relaxation curves when exposed to the oscillations and in the absence of stress is relatively insignificant [Figure 5A lower panel]. It is worthwhile to note that although the relaxation experiments were conducted with a specification that π remain constant close to 5 mN/m, this was found to be difficult for the OLA system, even though the same control software could successfully maintain constant surface pressure values for other materials (for example, fatty acids). Instead, for the OLA system, the surface pressure varied between 3 and 13 mN/m. However, it does not affect the monolayer stability, as can be seen in [Figure 5A lower panel]. Such high deviations are probably related to the bolaamphiphilic nature of OLA and the very stiff structure of the film [5,33]. As revealed using BAM, OLA monolayers in the form of tiles interacting with each other at the interface are susceptible to compression because of the very rigid structure. It is doubtful that excessively high values of the surface pressure achieved during oscillations and relaxations result from the feedback control loop utilized by the software. This is evidenced by the lack of such a phenomenon for other monolayers, such as, for example, oleic acid presented here.

Interestingly, even a minor addition of OA to the monolayer of OLA (OLA:OA 5:1) alters the trend of changes of viscoelastic moduli—over time, the E’ modulus decreases when E’’ stays at the steady level of ca. 100 mN/m [Figure 5B top panel]. The maximum value of the elastic modulus for this system was about 400 mN/m. The addition of even a small amount of the OA makes the monolayer more ductile. The monolayer of OLA:OA 5:1 is resistant even to strong stress such as barrier oscillations, so as a consequence, the relaxation during oscillation and without additional barrier movements follows the same course [Figure 5B lower panel]. Surface pressure amplitudes during the first few oscillations are as high as 11 mN/m, but in the initial stages of the relaxation experiment, they are at 5 ± 1 mN/m, and after 90 min, the monolayer becomes even more stable at 5 mN/m. Thus, we conclude that the addition of OA enhances the stability of the OLA monolayer.

Increasing the amount of OA in the mixture to OLA:OA 2:1 follows similar trends of the viscoelastic properties over time, but after 30 min the moduli stabilize [Figure 5C top panel]. Surprisingly for the equimolar system, we noted the rapid increase in E’ according to decreasing A/A_0_ [Figure 5D]. E’’ values are close to 0 mN/m until ca. 60 min. When the oscillation/relaxation curve stabilizes, so does the viscoelastic modulus. For this monolayer composition, E’ reaches the highest values of all the investigated systems (650 mN/m), but the π variations are the largest as well. A slight decrease in the A/A_0_ value can be observed [Figure 5D lower panel]. Almost ideal consistency among oscillation/relaxation and relaxation curves for described binary systems at 5 mN/m may be related to the attractive interactions between the molecules among the monolayer and relatively good miscibility of the components.

The behavior of the pure oleic acid monolayer is quite different—E’ and E’’ moduli are very small [Figure 5G top panel], and the area of monolayer coverage decreases very rapidly during the relaxation process [Figure 5G lower panel]. The maximal values of E’ and E’’ of the OA monolayer captured at the initial stage of the process equal 25 mN/m and 3 mN/m, respectively. For the film compositions in which OA predominates, E’ moduli reach values of 250 mN/m and 100 mN/m for OLA:OA 1:2 and 1:5, respectively, while E’’ reaches 35 mN/m and 15 mN/m [Figure 5E and F top panels]. In the initial stage of the experiment, the viscoelastic moduli are almost constant and then increase reaching the maximum. For OLA:OA 1:2 and 1:5 systems, surface pressure deviations are significantly limited and shifted after 60 and 150 min of the relaxation process [Figure 5E and F lower panels]. What is more, the oscillation cycles strongly affect the shape of the relaxation curves. We note large differences between basic relaxation and oscillation/relaxation curves, where A/A_0_ declines rapidly during the first 30 min of the measurement. This effect is even more pronounced for the pure OA monolayer—the barrier oscillations destabilized the monolayer and led to its large contraction after four oscillation cycles [Figure 5G lower panel]. The susceptibility to the stress caused by the barrier movement is due to the *cis* double bond in the hydrocarbon chain of the OA molecule.

## 4. Discussion

Among the tested systems, the OLA-rich mixture (5:1) is the only example where the surface pressure during the relaxations between oscillations is stable even over a long measurement time, and the monolayer is resistant to the oscillation-induced compression [Figure 5B lower panel]. Such behavior is qualitatively different from the pure OLA monolayer. Based on the evidence, we followed the theory of Brezesinski, Vollhardt, and Iimura [6], studying the interactions among oleanolic acid/stearic acid (SA) systems at the air/water interface, according to which the fatty acid molecules incorporate into the lattice of triterpenoid predominant quantitatively in the mixed monolayer. It was concluded that the addition of a small dose (10 mol%) of OLA to SA caused the phase separation, while the presence of 10 mol% SA in the OLA monolayer improved its compressibility. This effect is also noted for the mixed systems OLA:OA 2:1 and 5:1, in which compressibility is enhanced. Thus, this suggests that OA molecules integrate into the OLA lattice and favor the vertical orientation of the triterpenoid molecules. The values of the compression moduli for OLA:OA 5:1 and 2:1 are higher than for pure OLA monolayers. Therefore, it may be concluded that the orientation of OLA molecules towards the subphase is vertical, suggesting the –OH moieties are anchored in the water. Moreover, based on the BAM images of OLA:OA 5:1 in comparison with pure OLA (gained at the same experimental parameters and conditions), the relatively brighter and more homogeneous domains can be observed at corresponding surface pressures. This proves the presence of thicker structures (where the hydroxyl group faces the subphase), the axis of the molecule is perpendicular, and the monolayer is of greater height. In contrast to the system of OLA:OA 5:1, in other mixtures, the fluctuations increase over the measurement time. Such a phenomenon can be explained by the presence of the repulsive interactions between OLA and OA molecules and the phase separation. The extreme stiffness of the OLA film at the air/water interface is reflected in the enormous increase in π values (even up to 13 mN/m for the OLA:OA 1:1 system) [22]. On the other hand, the picture that OA molecules are excluded from these monolayers is also likely. This theory explains the stabilization of the relaxation curves for the immiscible systems and the increasing values of the viscoelastic moduli (OLA is characterized by higher E and E’’ than OA).

Fundamental research on interfacial properties is a powerful tool in predicting and explaining the behavior of specific pharmaceutical formulations. The occurrence of the miscibility gaps within the OLA-OA monolayers in the range of the molar compositions influences the applicability of binary systems. The previously mentioned investigations [16] on the preparation of NLCs loaded with oleanolic acid, and gentiopicrin with the oleic acid as a liquid lipid, revealed that the entrapment efficiency of OLA improves when OA content increases from 10 to 50%. The increase prevalence of OA affects the number of imperfections within the lipid matrix, and in consequence, more drug molecules could be trapped. The results of our research provide a reasonable explanation for such phenomena. The presence of numerous OA molecules in the OLA lattice causes system destabilization by the repulsive forces acting between the molecules of two substances, leading even to phase separation noticed as the system imperfections.

## 5. Conclusions

In summary, the biomimetic system of oleanolic and oleic acids at various molar ratios was tested at the interface, utilizing the Langmuir technique in terms of physicochemical characterization, miscibility, and rheology. The primary conclusion that can be drawn from this study is that the composition of the OLA/OA mixed monolayers determines the morphology, stability, and thermodynamic and viscoelastic properties of the binary systems.

It was found that numerous miscibility gaps are present for particular molar mixtures at various surface pressures. Most systems (OLA:OA 2:1, 1:1, 1:5) are considered to be immiscible at surface pressures >10 mN/m. Moreover, the OLA:OA 1:2 mixture is immiscible for every surface pressure tested. However, the system with the quantitative dominance of OLA to OA (5:1) emerged as the only one among the examined systems where interactions between molecules are more energetically favorable than for pure substances in the whole range of tested surface pressures.

The favorable characteristics of the OLA:OA 5:1 system are attributed to the incorporation of the OA molecules into the OLA 2D lattice and attractive interactions between the molecules. OA likely influences the orientation of the OLA molecules so that the hydroxyl group is directed towards the subphase. Such an arrangement significantly improves the stability of the mixed film. For the immiscible systems, when phase separation occurs, oleic acid molecules may be excluded from the monolayer.

We believe that our results may improve knowledge about interactions between components of the pharmaceutical formulations and be helpful in drug designing for triterpenoids, as well as other active substances, since the proper selection of carriers and excipients is crucial for stability improvement.

## Data Availability

Not applicable.

## Supplementary Materials

The following supporting information can be downloaded at: https://www.mdpi.com/article/10.3390/membranes12121215/s1, Figure S1: A schematic drawing of the oscillation-relaxation experiment. The experiment aimed to investigate the values of the E’ and E’ moduli over time. After monolayer compression to 5 mN/m, the consecutive oscillation cycles were separated by 10-minute intervals during which the surface pressure was kept at 5mN/m.; Figure S2: An example graph of the π-A isotherm (A) and the Cs^−1^ vs. π (B) with the characteristic values highlighted; Table S1: The characteristic values determined based on the π–A isotherms and compression moduli of OLA, OA and their mixtures; Figure S3: An example graph of the relative area A/A_0_ vs. time and the surface pressure vs. time, varying significantly due to the oscillation (A) and close-up emphasizing the peak asymmetry of the surface pressure plot.
