# Supplementary material for: The Biomimetic System of Oleanolic Acid and Oleic Acid at the Air-Water Interface–Interactions in Terms of Nanotechnology-Based Drug Delivery Systems
Source: Membranes (Basel). 2022 Dec 1;12(12):1215. doi: 10.3390/membranes12121215 (PMC9782233; doi:10.3390/membranes12121215)
Supplement: Supplementary file 1 [file membranes-12-01215-s001.zip › membranes-2043610-supplementary.pdf]

The Biomimetic System of Oleanolic Acid and Oleic Acid at the Air-Water Interface–Interactions in Terms of Nanotechnology-Based Drug Delivery Systems

Martyna Krajewska *, Katarzyna Dopierała * and Krystyna Prochaska

Institute of Chemical Technology and Engineering, Poznan University of Technology, Berdychowo 4, 60-965 Poznań, Poland
* Correspondence: martyna.m.krajewska@doctorate.put.poznan.pl (M.K.); katarzyna.dopierala@put.poznan.pl (K.D.); Tel.: +48-61-665-33-42 (M.K.); +48-61-665-37-72 (K.D.)

Supplementary materials

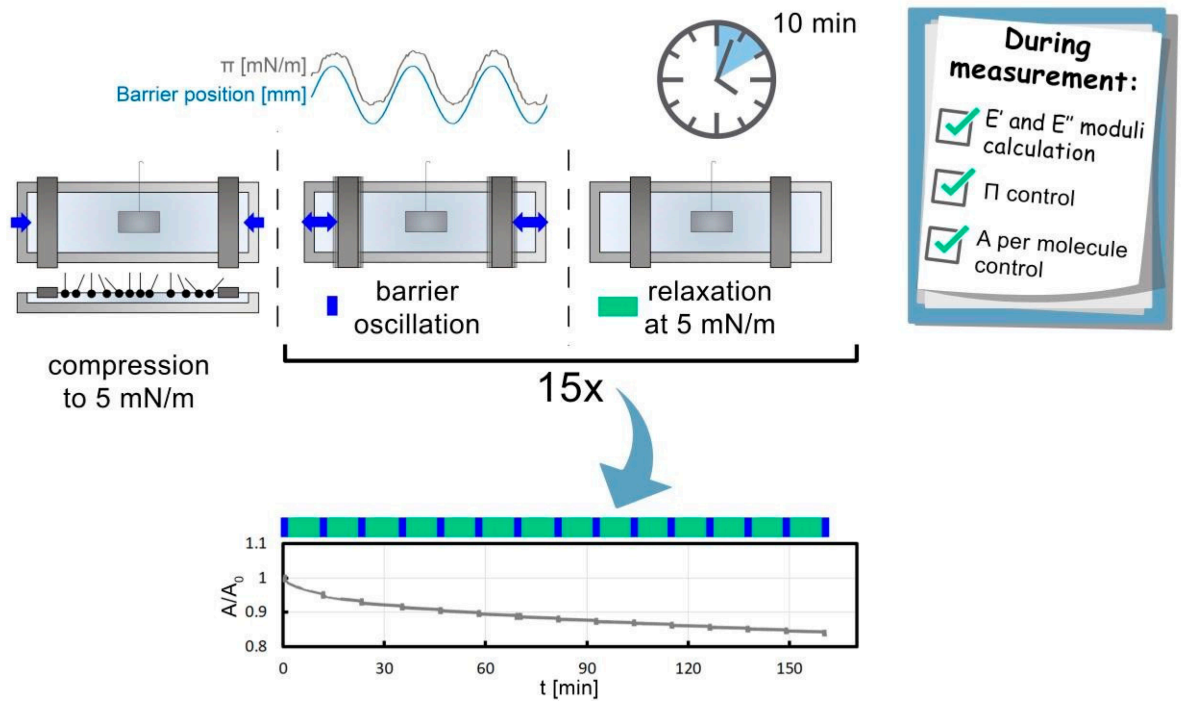

Figure S1. A schematic drawing of the oscillation-relaxation experiment. The experiment aimed to investigate the values of the E' and E' moduli over time. After monolayer compression to 5 mN/m, the consecutive oscillation cycles were separated by 10-minute intervals during which the surface pressure was kept at 5mN/m.

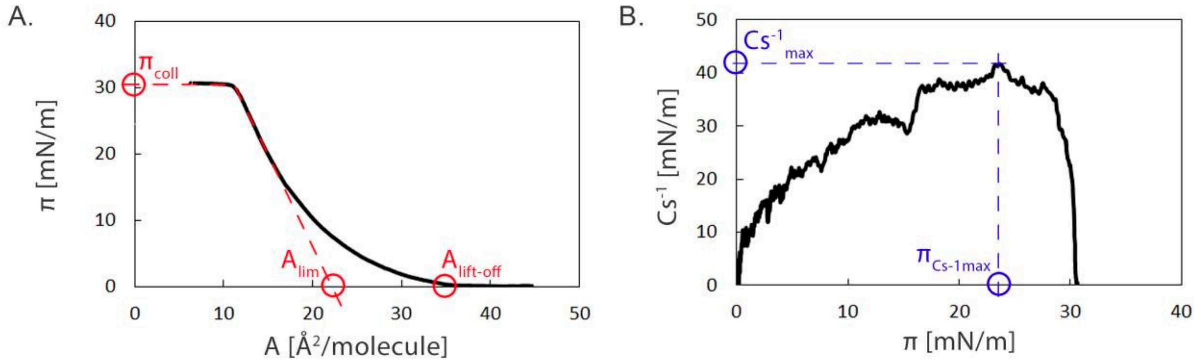

Figure S2. An example graph of the π-A isotherm (A) and the Cs^{-1} vs. π (B) with the characteristic values highlighted.

Table S1. The characteristic values determined based on the π – A isotherms and compression moduli of OLA, OA and their mixtures.

System	$A_{lift\text{-}off}$ Å²/molecule	A_{lim} Å²/molecule	π_{coll} mN/m	Cs^{-1}_{max} mN/m	ΠCs^{-1}_{max} mN/m	*State*
**OLA**	75	53	41	130	29	LC
**OLA:OA 5:1**	56	43	37	209	28.5	LC
**OLA:OA 2:1**	43	41	35	232	23.5	LC
**OLA:OA 1:1**	42	38	29	104	18	LC
**OLA:OA 1:2**	40	32	29	68	19.5	LE – LC
**OLA:OA 1:5**	37	25	29	99	25.5	LE – LC
**OA**	34	23	31	42	23.5	LE

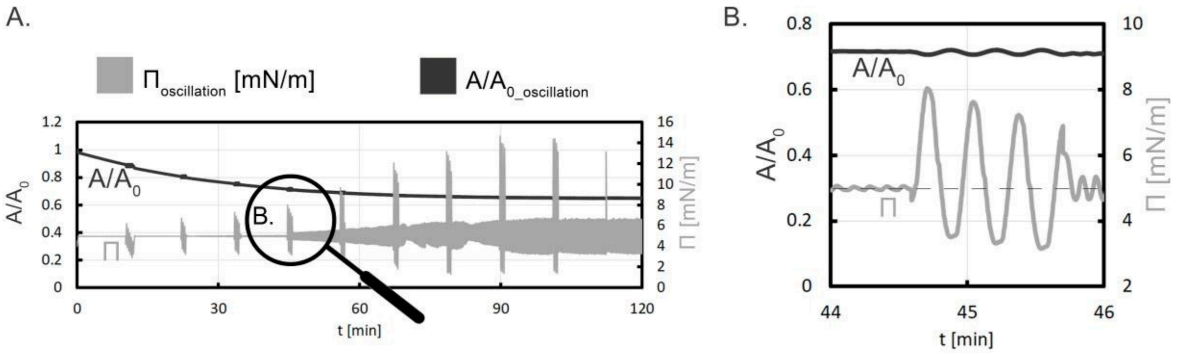

Figure S3. An example graph of the relative area A/A_0 vs. time and the surface pressure vs. time, varying significantly due to the oscillation (A) and close-up emphasizing the peak asymmetry of the surface pressure plot.
